# Risk stratification of cardiac arrhythmias and sudden cardiac death in type 2 diabetes mellitus patients receiving insulin therapy: A population‐based cohort study

**DOI:** 10.1002/clc.23728

**Published:** 2021-09-21

**Authors:** Sharen Lee, Kamalan Jeevaratnam, Tong Liu, Dong Chang, Carlin Chang, Wing Tak Wong, Ian Chi Kei Wong, Gregory Y. H. Lip, Gary Tse

**Affiliations:** ^1^ Diabetes Research Unit Cardiovascular Analytics Group, Hong Kong, China‐UK Collaboration China; ^2^ Faculty of Health and Medical Sciences University of Surrey Guildford UK; ^3^ Tianjin Key Laboratory of Ionic‐Molecular Function of Cardiovascular disease, Department of Cardiology, Tianjin Institute of Cardiology Second Hospital of Tianjin Medical University Tianjin China; ^4^ Xiamen Cardiovascular Hospital Xiamen University Xiamen China; ^5^ Division of Neurology, Department of Medicine Queen Mary Hospital Hong Kong China; ^6^ School of Life Sciences Chinese University of Hong Kong Hong Kong China; ^7^ Department of Pharmacology and Pharmacy University of Hong Kong Pokfulam China; ^8^ Medicines Optimisation Research and Education (CMORE) UCL School of Pharmacy London UK; ^9^ Liverpool Centre for Cardiovascular Science, University of Liverpool and Liverpool Heart & Chest Hospital, Liverpool, United Kingdom; and Department of Clinical Medicine Aalborg University Aalborg Denmark; ^10^ Kent and Medway Medical School Canterbury Kent UK

**Keywords:** cardiac arrhythmias, sudden cardiac death, type 2 diabetes

## Abstract

**Introduction:**

Metabolic abnormalities may exacerbate the risk of adverse outcomes in patients with type 2 diabetes mellitus. The present study aims to assess the predictive value of HbA1c and lipid variability on the risks of sudden cardiac death (SCD) and incident atrial fibrillation (AF).

**Methods:**

The retrospective observational study consists of type 2 diabetic patients prescribed with insulin, who went to publicly funded clinics and hospitals in Hong Kong between January 1, 2009 and December 31, 2009. Variability in total cholesterol, low‐density lipoprotein‐cholesterol (LDL‐C), high‐density lipoprotein‐cholesterol (HDL‐C), triglyceride, and HbA1c were assessed through their SD and coefficient of variation. The primary outcomes were incident (1) ventricular tachycardia/ventricular fibrillation, actual or aborted SCD and (2) AF.

**Results:**

A total of 23 329 patients (mean ± SD age: 64 ± 14 years old; 51% male; mean HbA1c 8.6 ± 1.3%) were included. On multivariable analysis, HbA1c, total cholesterol, LDL‐C and triglyceride variability were found to be predictors of SCD (*p* < .05).

**Conclusion:**

HbA1c and lipid variability were predictive of SCD. Therefore, poor glucose control and variability in lipid parameters in diabetic patients are associated with aborted or actual SCD. These observations suggest the need to re‐evaluate the extent of glycemic control required for outcome optimization.

## 1 INTRODUCTION

Sudden cardiac death (SCD) is a leading cause of death worldwide, accounting for approximately 25% of deaths of cardiovascular origin.^1^ By contrast, atrial fibrillation (AF), the most common sustained arrhythmia amongst adults, is increasingly prevalent around the world, particularly amongst developed countries.^2^, ^3^ Significant increases in morbidity and mortality amongst large‐scale epidemiological studies have been demonstrated amongst AF patients, including an increase in SCD risk.^4^, ^5^, ^6^, ^7^ Furthermore, type 2 diabetes mellitus increases the risk of SCD, as demonstrated by a recent meta‐analysis of population‐based prospective studies.^8^

With the global shift towards a more personalized approach in the management of diabetes, there is an increasing interest in exploring the application of new parameters, such as HbA1c and lipid variability, to better monitor disease progression and evaluate the prognosis. Although the exact mechanisms remain unclear, increased long‐term glycemic and lipid variability is hypothesized to lead to endothelial dysfunction via an increase in oxidative stress.^9^, ^10^, ^11^ Since hemoglobin has an average lifespan of 100 days, HbA1c can reflect glycemic control in recent months. Therefore, HbA1c variability is not affected by short‐term glycemic changes due to diet and medication changes, thus it is a better representation of long‐term glycemic variation. However, existing studies have focused on risk prediction of all‐cause mortality and general cardiovascular adverse events,^12^, ^13^, ^14^, ^15^, ^16^ with a limited number of studies exploring specifically arrhythmic risks amongst diabetics. Moreover, those type 2 diabetics who are partially or fully dependent on insulin are more likely to have severe disease and may be at higher risks of arrhythmias.

The aim of the present study was to assess the predictive value of HbA1c and lipid variability towards aborted or successful SCD, as well as incident AF in type 2 diabetic patients receiving insulin therapy.

## 2 METHODS

This study is a retrospective observational study approved by The Joint Chinese University of Hong Kong – New Territories East Cluster Clinical Research Ethics Committee (Application reference: 2018.462, 2018.643, 2019.361 [approval date: 15th August 2019]). The study cohort contains type 2 diabetic patients with insulin prescribed from any hospitals and outpatient clinics under the Hong Kong Hospital Authority from January 1, 2009 to December 31, 2009. The study cohort from 10 years ago was selected to ensure there is adequate follow‐up especially for rarer events such as ventricular arrhythmias and SCD. Clinical and biochemical data of eligible patients were obtained through the clinical data analysis and reporting system (CDARS), an electronic healthcare record database that integrates data across all publicly funded hospitals and clinics in Hong Kong. This system has been used by our team and other teams to conduct population‐based studies on different cardiovascular diseases,^17^, ^18^, ^19^, ^20^ including diabetes mellitus,^21^ in the past.

### 2.1 Patient data

Clinical and biochemical data of the present cohort were extracted from CDARS. The outcomes of the present study are the occurrence of SCD and AF from January 1, 2009 to December 31, 2019. SCD is defined as episodes of VT, VF, or nonspecific cardiac arrest, which were diagnosed under clinical judgment with electrocardiographic or biochemical findings and subsequently coded into hospital records. Both aborted and actual SCD events were included. VT/VF on electrophysiological study is not included. Hypoglycemia‐induced cases of SCD or AF were defined as cases with dextrose infusion during the admission episode or had blood glucose measured ≤3.9 mg/mmol. Demographic details, including age and sex, were extracted. Patients were categorized into four groups based on their age: below age 55, between ages 55 and 64, between ages 65 and 74, above and include age 75. The number of baseline acute hospitalization episodes between January 1, 2004 and December 31, 2008 was also obtained. Furthermore, the average daily dose of different classes of cardiovascular medications and antidiabetic agents was calculated by averaging the multiple between the daily dose frequency and drug dose by all patients with prescriptions of the specific drug class. Eight classes of antidiabetic agents were examined: Insulin, sulphonylurea, biguanide, alpha‐glucosidase inhibitor, thiazolidinedione, dipeptidyl peptidase‐4 inhibitor (DPP‐4I), glucagon‐like peptide receptor‐1 agonist (GLPA), and meglitinide. Data on five classes of cardiovascular drugs were obtained: angiotensinogen‐converting‐enzyme inhibitor/angiotensin receptor blocker (ACEI/ARB), beta‐adrenergic inhibitor, calcium channel blocker (CCB), diuretics, and lipid‐lowering agents

In terms of patient comorbidities, the number of nondiabetic comorbidities and diabetes‐related complications between January 1, 1999 and December 31, 2008 were obtained. The specific diabetic‐related complications recorded include: (1) Amyotrophy, (2) Arthropathy, (3) Hyperosmolar hyperglycemic state/diabetic ketoacidosis, (4) Hypoglycemia, (5) Neuropathy, (6) Retinopathy/maculopathy, (7) Peripheral vascular disease/angiopathy, and (8) Nephropathy. Patient's past medical history of the following conditions that initiated between January 1, 1999 and December 31, 2008 were also extracted: (1) Chronic kidney disease (CKD), (2) Chronic obstructive pulmonary disease (COPD), (3) Chronic liver disease (CLD), (4) Heart failure (HF), (5) Ischemic heart disease (IHD), (6) Hypertension, (7) Acute myocardial infarction, and (8) Stroke. International classification of disease, ninth edition (ICD‐9) codes was used to extract the study outcomes and pre‐existing comorbidities (Supplementary Table 1), whilst the diabetic complications were extracted using the ICD‐9 based hospital authority master disease code table for greater specificity.

Baseline data of urinalysis, renal and liver function tests, complete blood count, and other blood tests within the year 2008 were extracted. Urinalysis results include: (1) Urine albumin/creatinine ratio, (2) Creatinine clearance, (3) 24‐hours total urine protein and albumin, (4) Spot urine protein, albumin, and glucose. Indices from complete blood count include: (1) The absolute number of hemoglobin, basophil, eosinophil, platelet, red and white blood cell, (2) Mean corpuscular volume, (3) Mean corpuscular hemoglobin concentration, (4) Mean corpuscular hemoglobin (MCH), (5) Hematocrit. The presence of anaemia, defined by sex‐based thresholds of below 13 g/dl for male and 12 g/dl for female, were obtained. Blood test results extracted include: (1) Serum creatinine, (2) Serum sodium and potassium, (3) Serum urea and urate, (4) Total serum protein and albumin, (5) Total serum bilirubin, alanine aminotransferase and alkaline phosphatase, (6) Fasting and random blood glucose. The presence of hypoglycemia at baseline and frequency of hypoglycemic episodes were extracted. Hypoglycemia was defined by fasting or random blood glucose below 3.9 mg/mmol.

The following blood results between January 1, 2004 and December 31, 2008 were extracted for the evaluation of their mean, variability and baseline value: (1) Total cholesterol, (2) High‐density lipoprotein cholesterol (HDL‐C), (3) Low‐density lipoprotein cholesterol (LDL‐C), (4) Total triglyceride, and (5) HbA1c. LDL‐C results included findings from both direct and calculated measurements. Variability analysis of a biochemical index was only executed on patients with at least three measurements.

### 2.2 Statistical analysis

Statistical analysis was performed using R Studio, and statistical significance was defined as *p* value <.05. Kaplan–Meier survival curve was used to portray the difference in actual or aborted SCD and AF survival between patients of different age groups, with the statistical significance of the intergroup difference evaluated using the log‐rank test. Temporal variability of HbA1c and lipid indices were evaluated using calculated parameters of SD and coefficient of variation (CV). CV was measured by multiplying 100‐fold of the value calculated by the ratio SD and mean. SD and CV were used to measure variability since they were less affected by outliers. Whilst SD is independent of the mean, CV is independent of the scale thus more sensitive to small changes to the mean. To identify predictors for shorter time to aborted or actual SCD and AF occurrence, univariable Cox regression was first applied to clinical and biochemical parameters. Patients with missing data were excluded from the analysis. Furthermore, due to their limited prescription towards the study cohort, GLPA and meglitinide were not included. Subsequently, parameters with *p* value <0.10 were included in the multivariable Cox regression model. Only patients with no missing data for the selected parameters, and at least three measurements for the selected variability predictors, were included in the multivariate models. No data imputation was performed.

The inter‐relations between HbA1c and lipid variability with intermittent hypoglycemia were evaluated using logistic and Poisson regression. Logistic regression was also used to examine the relationship between baseline hypoglycemia frequency and (1) occurrence of SCD/AF or (2) aborted or actual SCD/AF episodes that were associated with hypoglycemia. Odds ratios (ORs) were reported from logistic and Poisson regression, whereas hazard ratios were reported from Cox regression, along with the 95% confidence intervals.

## 3 RESULTS

**FIGURE 1:**
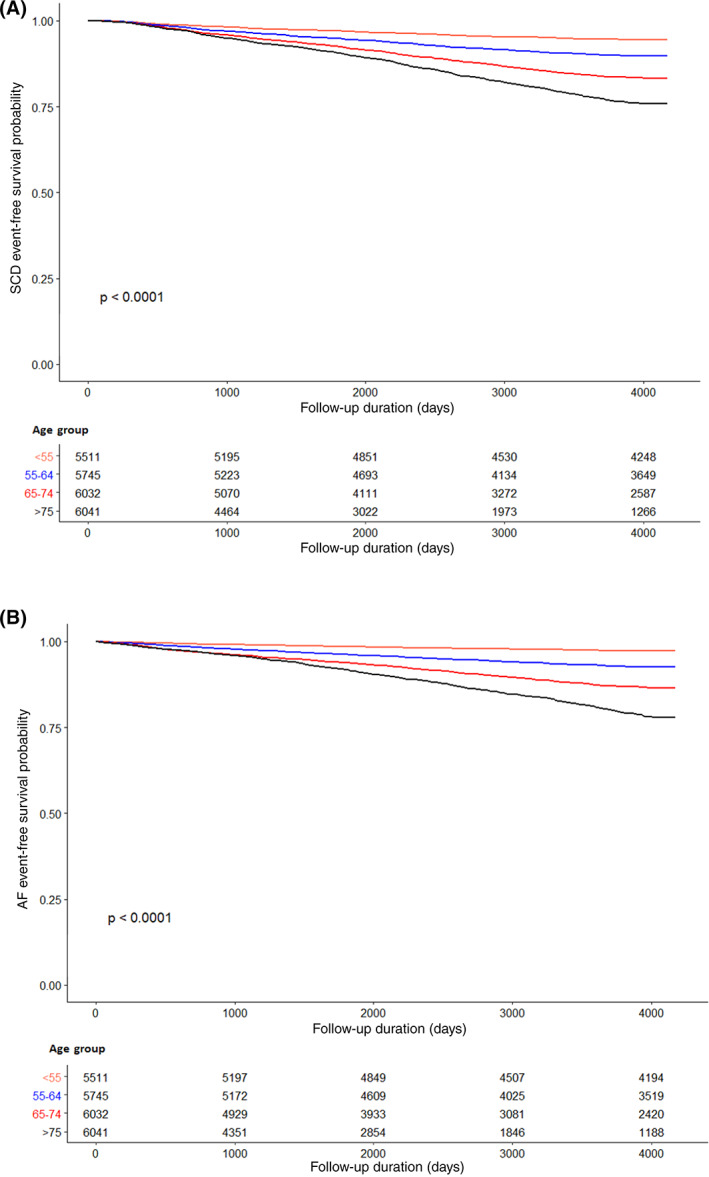
(A) Kaplan–Meier survival curves of different age groups for sudden cardiac death. (B) Kaplan–Meier survival curves of different age groups for atrial fibrillation

The present study included 23 329 patients (mean age = 64.3 ± 13.8, male = 50.8%, average mean HbA1c = 8.6 ± 1.3%, all‐cause mortality = 50.5%). The cohort was divided into four age groups: <55 (*n* = 5511), 55–64 (*n* = 5745), 65–74 (*n* = 6032), >75 (*n* = 6041), with a significant intergroup difference in survival for both aborted or actual SCD (*p* < .0001) and incident AF (*p* < .0001; Figure 1A, B). The baseline clinical and biochemical characteristics of the cohort are summarized in Tables 1 and 2 respectively. Patients had an average of 8.67 ± 7.81 distinct nondiabetic comorbidities, and 0.66 ± 0.90 diabetic complications. On average, patients had a median of three (interquartile range = 5) episodes of acute hospital admissions between the years 2004 and 2008. The three commonest diabetic complications were retinopathy/maculopathy (16.5%), nephropathy (14.7%), and hypoglycemia (11.9%), as shown in Table 1. Other pre‐existing comorbidities were hypertension (36.6%), IHD (16.7%), stroke (12.1%), HF (10.1%), CKD (9.0%), CLD (5.7%), AMI (5.3%), and COPD (3.4%). The 43.8% patients (*n* = 10 223, mean daily dose = 95.0 ± 300 mg/day) were on lipid‐lowering agents over the course of follow up.

**TABLE 1:** Baseline clinical characteristics

	*n*	%
Male	11 842	50.8
Comorbidities		
Chronic kidney disease	2099	9.00
Chronic obstructive pulmonary disease	783	3.36
Heart failure	2355	10.1
Ischemic heart disease	3904	16.7
Hypertension	8538	36.6
Chronic liver disease	1335	5.72
Acute myocardial infarction	1224	5.25
Stroke	2826	12.1
Pre‐existing diabetic complications		
Amyotrophy	70	0.300
Arthropathy	20	0.086
Hyperosmotic Hyperglycemic State/diabetic ketoacidosis	1267	5.43
Hypoglycemia	2787	11.9
Neuropathy	1339	5.74
Retinopathy/maculopathy	3848	16.5
Peripheral vascular disease/angiopathy	544	2.33
Nephropathy	3420	14.7

**TABLE 2:** Baseline biochemical characteristics

	Mean	SD
Urinalysis		
Albumin/creatinine ratio (mg/mmol)	40.3	125
Creatinine clearance (ml/min)	52.9	35.2
Spot protein (g/d)	1.19	1.96
Spot albumin (mg/L)	179	555
Spot glucose (mmol/L)	12.5	6.54
24‐hours total protein (g/d)	1.20	1.99
24‐hours total albumin (mg/d)	279	677
Baseline blood test		
Fasting glucose (mmol/L)	8.90	3.66
Random glucose (mmol/L)	12.2	7.36
HbA1c (%)	8.56	1.93
Total cholesterol (mmol/L)	4.74	1.12
High‐density lipoprotein (HDL) cholesterol (mmol/L)	1.22	0.387
Calculated low‐density lipoprotein (LDL) cholesterol (mmol/L)	2.74	0.930
Direct LDL cholesterol (mmol/L)	2.81	0.924
Triglyceride (mmol/L)	1.83	1.74
Thyroid‐stimulating globulin (TSH) (mIU/L)	2.28	4.13
Free thyroxine (fT4) (pmol/L)	14.7	37.3
Renal function test		
Creatinine (umol/L)	146	160
Sodium (mmol/L)	139	3.33
Potassium (mmol/L)	4.31	0.507
Urate (umol/L)	0.412	0.128
Urea (mmol/L)	8.98	6.11
Liver function test		
Albumin (g/L)	39.1	5.53
Alanine aminotransferase (ALT) (U/L)	24.3	21.0
Alkaline phosphatase (ALP) (U/L)	84.5	45.8
Total bilirubin (umol/L)	11.2	9.07
Total protein (g/L)	74.4	7.14
Complete blood count		
Hemoglobin (g/dl)	12.5	1.99
Mean corpuscular hemoglobin (MCH) (pg)	29.7	2.95
Mean corpuscular hemoglobin concentration (MCHC) (g/dl)	34.0	0.953
Mean corpuscular volume (MCV) (fL)	87.2	7.42
Hematocrit (L/L)	0.376	0.559
Basophil (×10^9^/L)	0.029	0.042
Eosinophil (×10^9^/L)	0.225	0.236
Lymphocyte (×10^9^/L)	1.87	0.866
Monocyte (×10^9^/L)	0.539	0.266
Neutrophil (×10^9^/L)	5.46	2.72
Platelet (×10^9^/L)	255	83.4
Red blood cell (×10^12^/L)	4.25	0.740
White blood cell (×10^9^/L)	8.09	2.86

The baseline biochemical characteristics of the present cohort are presented in Table 2. The average frequency of baseline hypoglycemic episodes, between January 1 and December 31 2008, was 0.50 ± 1.31 episodes per year. The baseline hypoglycemia frequency significantly correlated with all HbA1c and lipid variability indices (*p* < .0001). Logistic regression revealed that baseline hypoglycemia frequency was a significant predictor of aborted or actual SCD (OR = 1.09, 95% CI = [1.06, 1.12], *p* < .0001) and incident AF (OR = 1.05, 95% CI = [1.01, 1.08], *p* = .007). In total, 2512 and 1846 patients experienced incident SCD and AF respectively throughout follow‐up.

### 3.1 Predictors of SCD and AF

In the present cohort, 10.3% of patients suffered from at least one SCD event, and 7.7% suffered from AF. Amongst these cases, 25 and 10 cases of patients experiencing aborted or actual SCD and AF were associated with hypoglycemia on admission, respectively. Supplementary Table 2 and 3 present the univariable Cox regression of predictors for aborted or actual SCD and AF respectively. Supplementary Table 4 presents the univariable Cox regression for ventricular tachycardia alone, with HbA1c and cholesterol variability found to be predictive (*p* < .05).

Table 3 summarizes the results of the multivariable analysis for aborted or actual SCD (n = 15 316), where the following predictors were independent predictors: (1) Demographics: Age (HR = 1.04, 95% CI = [1.02, 1.05], *p* < .0001), male (HR = 1.27, 95% CI = [1.15, 1.41], *p* < .0001); (2) Clinical: Frequency of baseline acute admissions (HR = 1.003, 95% CI = [1.003, 1.003], *p* < .0001), number of concomitant diabetic complications (HR = 1.21, 95% CI = [1.15, 1.27], *p* < .0001), number of discrete nondiabetic comorbidities (HR = 1.02, 95% CI = [1.01, 1.02], *p* < .0001), hypoglycemic frequency (HR = 1.03, 95% CI = [1.00, 1.07], *p* = .032); (3) Glycemic and lipid variability: HbA1c SD (HR = 1.74, 95% CI = [1.45, 2.09], *p* < .0001) and CV (HR = 0.953, 95% CI = [0.935, 0.972], *p* < .0001), total cholesterol CV (HR = 1.05, 95% CI = [1.02, 1.08], *p* = .002), LDL‐C SD (HR = 1.73, 95% CI = [1.10, 2.74], *p* = .018), triglyceride mean (HR = 1.29, 95% CI = [1.17, 1.42], *p* < .0001) and CV (HR = 1.01, 95% CI = [1.00, 1.01], *p* = .022); (4) Antidiabetic agent: Sulphonylurea (HR = 1.39, 95% CI = [1.25, 1.54], *p* < .0001), biguanide (HR = 0.630, 95% CI = [0.563, 0.704], *p* < .0001); (5) Cardiovascular medications: Beta‐blocker (HR = 1.24, 95% CI = [1.12, 1.38], *p* < .0001), diuretic (HR = 1.51, 95% CI = [1.35, 1.67], *p* < .0001). CV of HbA1c (HR = 0.953, 95% CI = [0.935, 0.972], *p* < .0001) and LDL‐C (HR = 0.978, HR = [0.966, 0.991], *p* = .001), in addition to SD of total cholesterol (HR = 0.551, 95% CI = 0.275, 0.951), *p* = .034) and triglyceride (HR = 0.772, 95% CI = [0.656, 0.909], *p* = .002) were predictive of SCD on univariable analysis but became protective after multivariable analysis.

**TABLE 3:** Multivariable analysis showing predictors of sudden cardiac death (*n* = 15 316)

Predictor	Hazard ratio (HR)	95% confidence interval (CI)	*p* value
Age	1.04	[1.02, 1.05]	**<.0001**
Categorized age	0.977	[0.846, 1.13]	0.747
Male	1.27	[1.15, 1.41]	**<.0001**
Frequency of baseline acute admissions	1.003	[1.003, 1.003]	**<.0001**
Number of concomitant DM complications	1.21	[1.15, 1.27]	**<.0001**
Number of distinct non‐DM comorbidities	1.02	[1.01, 1.02]	**<.0001**
Baseline anemia	0.948	[0.861, 1.04]	0.281
Hypoglycemia frequency	1.03	[1.00, 1.07]	**.032**
HbA1c			
SD	1.74	[1.45, 2.09]	**<.0001**
Coefficient of variation	0.953	[0.935, 0.972]	**<.0001**
Total cholesterol			
SD	0.551	[0.275, 0.951]	**.034**
Coefficient of variation	1.05	[1.02, 1.08]	**.002**
HDL cholesterol			
Mean	0.866	[0.609, 1.23]	0.425
SD	0.915	[0.120, 6.99]	0.931
Coefficient of variation	1.00	[0.979, 1.03]	0.769
LDL cholesterol			
SD	1.73	[1.10, 2.74]	**.018**
Coefficient of variation	0.978	[0.966, 0.991]	**.001**
Triglyceride			
Mean	1.29	[1.17, 1.41]	**<.0001**
SD	0.772	[0.656, 0.909]	**.002**
Coefficient of variation	1.006	[1.00, 1.01]	**.022**
Antidiabetic agent			
Sulphonylurea	1.39	[1.25, 1.54]	**<.0001**
Biguanide	0.630	[0.563, 0.704]	**<.0001**
Thiazolidinedione	0.849	[0.652, 1.11]	0.226
Cardiovascular medications			
ACEI/ARB	1.02	[0.914, 1.14]	0.724
Beta‐blocker	1.24	[1.12, 1.38]	**<.0001**
Calcium channel blocker	1.17	[1.06, 1.30]	.002
Diuretic	1.51	[1.35, 1.67]	**<.0001**
Lipid‐lowering agents	1.02	[0.915, 1.14]	0.697

*Note*: Values in bold indicate *P* < 0.05.

The findings of multivariable regression analysis for incident AF (*n* = 13 267) are presented in Table 4, where several significant predictors were identified: (1) Demographics: Age (HR = 1.04, 95% CI = [1.03, 1.06], *p* < .0001); (2) Clinical: Frequency of baseline acute admissions (HR = 1.00, 95% CI = [1.00, 1.00], *p* < .0001), hypoglycemic frequency (HR = 1.05, 95% CI = [1.02, 1.09], *p* = .005); (3) Antidiabetic agent: Sulphonylurea (HR = 1.12, 95% CI = [1.00, 1.26], *p* = .043), biguanide (HR = 0.843, 95% CI = [0.746, 0.954], *p* = .007); (4) Cardiovascular medication: ACEI/ARB (HR = 1.21, 95% CI = 1.06, 1.37), *p* = .004), beta‐blocker (HR = 1.56, 95% CI = [1.39, 1.75], *p* < .0001), CCB (HR = 1.35, 95% CI = [1.20, 1.51], *p* < .0001), diuretic (HR = 1.50, 95% CI = [1.34, 1.69], *p* < .0001), lipid‐lowering agents (HR = 1.16, 95% CI = [1.03, 1.32], *p* = .018).

**TABLE 4:** Multivariable analysis showing predictors of atrial fibrillation (*n* = 13 267)

Predictor	Hazard ratio (HR)	95% confidence interval (CI)	*p* value
Age	1.04	[1.03, 1.06]	**<.0001**
Categorized age	1.09	[0.927, 1.27]	0.307
Male	0.916	[0.816, 1.03]	0.131
Frequency of baseline acute admissions	1.002	[1.002, 1.003]	**<.0001**
Number of concomitant DM complications	1.03	[0.961, 1.09]	0.437
Number of distinct non‐DM comorbidities	0.998	[0.991, 1.01]	0.700
Hypoglycemia frequency	1.05	[1.02, 1.09]	**.005**
HbA1c			
Mean	1.00	[0.927, 1.08]	0.973
SD	1.31	[0.898, 1.92]	0.161
Coefficient of variation	0.987	[0.954, 1.02]	0.474
Total cholesterol			
Mean	1.10	[0.800, 1.50]	0.565
SD	1.05	[0.522, 2.11]	0.893
Coefficient of variation	0.997	[0.961, 1.03]	0.894
HDL cholesterol			
Mean	0.907	[0.580, 1.42]	0.668
SD	0.296	[0.030, 2.95]	0.299
Coefficient of variation	1.02	[0.993, 1.05]	0.143
LDL cholesterol			
Mean	0.783	[0.566, 1.08]	0.142
SD	1.36	[0.730, 2.55]	0.331
Coefficient of variation	0.992	[0.976, 1.01]	0.340
Triglyceride			
Mean	0.968	[0.860, 1.09]	0.589
Antidiabetic agent			
Sulphonylurea	1.12	[1.00, 1.26]	**.043**
Biguanide	0.843	[0.746, 0.954]	**.007**
Cardiovascular medications			
ACEI/ARB	1.21	[1.06, 1.37]	**.004**
Beta‐blocker	1.56	[1.39, 1.75]	**<.0001**
Calcium channel blocker	1.35	[1.20, 1.51]	**<.0001**
Diuretic	1.50	[1.34, 1.69]	**<.0001**
Lipid‐lowering agents	1.16	[1.03, 1.32]	**.018**

*Note*: Values in bold indicate *P* < 0.05.

## 4 DISCUSSION

The present study demonstrated the following major findings: (1) Clinical and biochemical indices are predictive of arrhythmic occurrence amongst diabetics; (2) Both the mean and variability of HbA1c and lipid indices can predict VT/VF/SCD in diabetic patients; (3) HbA1c variability is associated with hypoglycemia frequency. To the best of our knowledge, the present study is the first to report an association between increased variability in HbA1c and lipid markers with increased risk for VT/VF/SCD amongst diabetic patients.

The prognostic values of HbA1c and lipid variability have been increasingly explored over the past decades. However, prior studies have mostly focused on the prediction of all‐cause mortality or cardiovascular events^12^, ^22^ with only a limited number of studies focusing on arrhythmic or SCD outcomes.^23^ Although the underlying pathophysiology remains incompletely elucidated, there are several possible contributing factors towards the increased arrhythmic risk amongst patients with high glycemic and lipid variability. First, increased glycemic variability is found to be associated with QTc prolongation and increased QTc dispersion, which greatly elevates the risk of ventricular tachyarrhythmia.^24^, ^25^ There is evidence suggesting that QTc prolongation may be triggered by spontaneous hypoglycemia due to underlying coronary atherosclerosis or cardiac autonomic neuropathy.^26^, ^27^, ^28^, ^29^ Antidiabetic agent use may also play a role in the prognostic value of glycemic variability. Biguanide users are likely more stable or earlier in the disease course, thus have a lower cardiovascular disease burden. Sulphonylurea use, which was predictive of SCD in the present study, is known to have an increased risk of hypoglycemia.^30^ In addition, it has been reported human ether‐a‐go‐go‐related gene (hERG) channel inhibitory effects of some sulphonylurea, which can lead to QT prolongation.^31^ Amongst patients on insulin, who have more labile glucose control, the spontaneous glycemic fluctuations can induce the occurrence of arrhythmia. Unfortunately, continuous blood glucose monitoring was not available in the present study to demonstrate the association between spontaneous glycemic changes and arrhythmic episodes.

Furthermore, structural remodeling as a result of chronic hyperglycemia is also involved in the pathogenesis.^32^, ^33^, ^34^, ^35^, ^36^, ^37^ HbA1c variability has been associated with remodeling and fibrosis of the atria and ventricles, which could be arrhythmogenic.^38^, ^39^, ^40^ In a recent nationwide observational study, an association between high lipid variability and increased risk of new‐onset AF was reported, and statins protected against AF development via the reduction in adverse atrial remodeling.^41^ Moreover, frequent intermittent hypoglycemia can induce the release of reactive oxygen species,^42^ thereby leading to increased oxidative stress, chronic inflammation, and endothelial dysfunction.^43^ Hypoglycemia itself is arrhythmogenic and can reduce the myocardial tolerance to ischemia and reperfusion injuries.^44^, ^45^

Similar to glycemic variability, the increase in oxidative stress with fluctuations in lipid levels due to atherogenic substance release from unstable plaque is hypothesized to underlie the increased arrhythmic risk.^12^ Indeed, glycemic fluctuations were found to increase the formation of atherosclerotic plaques and thinning the fibrous cap, which suggests that intermittent hypoglycemia may contribute to lipid variability as well.^46^ It should be noted that whilst triglyceride and HDL‐C variability are dependent on glycemic control and other lifestyle factors, the use of statin plays a significant role in LDL‐C variability. The significant interpersonal variability, as well as the varying effects between different types of statins on LDL‐C variability, reflects the need for further research on the area.^47^, ^48^

The change in HbA1c and lipid variability from predictive of SCD, in univariable analysis, to being protective under multivariable analysis, can be attributed to several causes. The limitation of cohort size and multivariate analysis may have selected for patients of more advanced disease and undergone aggressive control. Given that a J‐shaped association between adverse outcome and both glycemic and lipid indices have been described, patients with high variability that returned to the optimal glycemic and lipid range would have had a better prognosis.^16^, ^49^, ^50^ Indeed, the duration of exposure to an optimal glycemic range is inversely associated with diabetic retinopathy progression, even after accounting for the effects of glycemic variability.^51^ Additionally, Ceriello et al. reported that patients with elevations in both HbA1c and HDL‐C variability were at higher risk for diabetic nephropathy than those with high variability in only one variable, highlighting the interacting effects between variability markers.^52^ Therefore, the protective value may be a result of inevitable selection bias, the protective effects of pharmacotherapy, and the interactions between different indices.^53^, ^54^

### 4.1 Strengths and limitations

There are four major strengths for the present study: (1) The independent, and interdependent predictive effects of clinical and biochemical indices towards SCD and AF were assessed by univariable and multivariable analysis; (2) The predictive values of both the value and variability of HbA1c and lipid indices were assessed to examine the effect of biochemical fluctuations on arrhythmic risk in diabetics; (3) The inter‐relationship between intermittent hypoglycemia, HbA1c and lipid variability and chronic inflammation was examined to elucidate the underlying pathogenic mechanism; (4) Long follow‐up durations permitted the capture of adverse outcomes over a long period.

However, several limitations should be recognized. Firstly, the cohort was limited to type 2 diabetic patients prescribed with insulin, which can limit the generalizability of the findings. Given that insulin is only prescribed for diabetic patients in later stages, an advanced disease state can be inferred for the selected patients. Secondly, the observational nature of the present study leads to inevitable errors from missing data, coding errors, and under‐coding. A causational relationship cannot be established from the findings of the observational study, which can only demonstrate associations. Furthermore, unfortunately, ICD coding does not reflect the frequency of events, thus the frequency of SCD events was not evaluated. The coding also does not reflect whether the VT is sustained or associated with hemodynamic collapse. It is based on the assumption that coded VT is clinically significant as nonsustained VT would be clinically irrelevant thus not coded into the database. It should be emphasized that a diagnosis of VT is not the same as SCD, hence the effect of long‐term glycemic variability on the risk of SCD should be interpreted with caution. Novel therapies, such as GLPA (*n* = 9) and SGLT‐2 transporter inhibitor (*n* = 0), were not assessed due to the fact that this retrospective study recruited patients in the year 2009, at which these agents were not yet developed. Finally, data on blood pressure, body mass index, echocardiogram, severity of HF and lifestyle were absent, which can affect the patients' cardiovascular health.

## 5 CONCLUSION

Poor glucose control and variability in lipid parameters in diabetic patients are associated with SCD. These observations suggest the need to re‐evaluate the extent of glycemic control required for outcome optimization. Further studies on the predictive value of variability in other glycemic measures, such as fasting and random blood glucose, in addition to other methods of measuring variability, should be performed to further examine the predictiveness of glycemic variability towards arrhythmias in diabetic patients.

## CONFLICTS OF INTEREST

No conflicts of interest.

## Supporting information


**Supplementary Table 1** ICD‐9 Codes for Outcomes and Comorbidities.
**Supplementary Table 2.** Univariable Predictors for Sudden Cardiac Deathz.
**Supplementary Table 3.** Univariable Predictors for Atrial Fibrillation.
**Supplementary Table 4.** Univariable Predictors for Ventricular Tachycardia.

## Data Availability

The deidentified dataset arising from this study is available upon request.
