# Supplementary material for: Risk stratification of cardiac arrhythmias and sudden cardiac death in type 2 diabetes mellitus patients receiving insulin therapy: A population‐based cohort study
Source: Clin Cardiol. 2021 Sep 21;44(11):1602–12. doi: 10.1002/clc.23728 (PMC8571559; doi:10.1002/clc.23728)
Supplement: Supplementary file 1 — Supplementary Table 1 ICD‐9 Codes for Outcomes and Comorbidities. Supplementary Table 2. Univariable Predictors for Sudden Cardiac Deathz. Supplementary Table 3. Univariable Predictors for Atrial Fibrillation. Supplementary Table 4. Univariable Predictors for Ventricular Tachycardia. [file CLC-44-1602-s001.docx]

**Supplementary Table 1.** ICD-9 Codes for Outcomes and Comorbidities.

|  | ICD Code |
| --- | --- |
| Outcome | |
| Atrial Fibrillation | 427.31, 429.4 |
| Sudden Cardiac Death | 427.1, 427.4, 427.5 |
| Pre-existing Comorbidity | |
| Chronic Renal Disease | 585.00-585.99 |
| Chronic Obstructive Pulmonary Disease | 490.00-496.99 |
| Heart Failure | 398.91, 402.91, 428.00-428.99 |
| Ischemic Heart Disease | 411.00-411.99, 413.00-414.99 |
| Hypertension | 401.9 |
| Chronic Liver Disease | 570.00-573.99 |
| Acute Myocardial Infarction | 410.00-410.99, 412.00-412.99 |
| Stroke | 430.00-438.99 |

**Supplementary Table 2.** Univariable Predictors for Sudden Cardiac Death.

| Predictor | Hazard Ratio (HR) | 95% Confidence Interval (CI) | P-Value |
| --- | --- | --- | --- |
| Age | 1.046 | [1.04, 1.05] | **< 0.0001** |
| Categorized Age | 1.61 | [1.55, 1.67] | **<0.0001** |
| Male | 1.27 | [1.17, 1.37] | **<0.0001** |
| Frequency of Baseline Acute Admissions | 1.005 | [1.00, 1.01] | **<0.0001** |
| Number of Concomitant DM Complications | 1.45 | [1.39, 1.51] | **<0.0001** |
| Number of Distinct Non-DM Comorbidities | 1.053 | [1.05, 1.06] | **<0.0001** |
| Baseline Hemoglobin Count | 1.01 | [0.989, 1.03] | 0.367 |
| Baseline Anemia | 0.930 | [0.854, 1.01] | 0.094 |
| Hypoglycemia Frequency | 1.11 | [1.08, 1.13] | **<0.0001** |
| HbA1c (n=20874) |  |  |  |
| Baseline | 1.00 | [0.981, 1.02] | 0.870 |
| Mean | 0.997 | [0.965, 1.03] | 0.834 |
| Standard Deviation | 1.10 | [1.06, 1.15] | **<0.0001** |
| Coefficient of Variation | 1.01 | [1.006, 1.02] | **<0.0001** |
| Total Cholesterol (n=18926) |  |  |  |
| Baseline | 0.973 | [0.938, 1.01] | 0.154 |
| Mean | 1.02 | [0.973, 1.08] | 0.362 |
| Standard Deviation | 1.33 | [1.25, 1.41] | **<0.0001** |
| Coefficient of Variation | 1.03 | [1.02, 1.03] | **<0.0001** |
| HDL Cholesterol (n=17930) |  |  |  |
| Baseline | 1.00 | [0.902, 1.12] | 0.943 |
| Mean | 0.471 | [0.406, 0.547] | **<0.0001** |
| Standard Deviation | 2.54 | [1.66, 3.90] | **<0.0001** |
| Coefficient of Variation | 1.024 | [1.02, 1.03] | **<0.0001** |
| LDL Cholesterol (n=17485) |  |  |  |
| Baseline | 1.01 | [0.962, 1.06] | 0.746 |
| Mean | 0.990 | [0.929, 1.06] | 0.749 |
| Standard Deviation | 1.66 | [1.48, 1.85] | **<0.0001** |
| Coefficient of Variation | 1.015 | [1.01, 1.02] | **<0.0001** |
| Triglyceride (n=18889) |  |  |  |
| Baseline | 1.01 | [0.991, 1.03] | 0.251 |
| Mean | 1.08 | [1.06, 1.10] | **<0.0001** |
| Standard Deviation | 1.05 | [1.02, 1.08] | **0.001** |
| Coefficient of Variation | 1.00 (1.004) | [1.00, 1.01] | **0.001** |
| Anti-Diabetic Agent |  |  |  |
| Sulphonylurea | 1.18 | [1.09, 1.28] | **<0.0001** |
| Biguanide | 0.464 | [0.428, 0.503] | **<0.0001** |
| DPP4 Inhibitor | 0.587 | [0.293, 1.18] | 0.132 |
| Thiazolidinedione | 0.663 | [0.519, 0.847] | **0.001** |
| Alpha-Glucosidase Inhibitor | 1.10 | [0.884, 1.37] | 0.393 |
| Cardiovascular Medications |  |  |  |
| ACEI/ARB | 1.23 | [1.13, 1.34] | **<0.0001** |
| Beta-blocker | 1.84 | [1.69, 1.99] | **<0.0001** |
| Calcium channel blocker | 1.97 | [1.82, 2.14] | **<0.0001** |
| Diuretic | 2.63 | [2.43, 2.85] | **<0.0001** |
| Lipid-lowering agents | 1.39 | [1.28, 1.50] | **<0.0001** |

**Supplementary Table 3.** Univariable Predictors for Atrial Fibrillation.

| Predictor | Hazard Ratio (HR) | 95% Confidence Interval (CI) | P-Value |
| --- | --- | --- | --- |
| Age | 1.059 | [1.05, 1.06] | **<0.0001** |
| Categorized Age | 1.86 | [1.77, 1.95] | **<0.0001** |
| Male | 0.903 | [0.823, 0.991] | **0.031** |
| Frequency of Baseline Acute Admissions | 1.004 | [1.00, 1.01] | **<0.0001** |
| Number of Concomitant DM Complications | 1.20 | [1.13, 1.27] | **<0.0001** |
| Number of Distinct Non-DM Comorbidities | 1.034 | [1.03, 1.04] | **<0.0001** |
| Baseline Hemoglobin Count | 0.981 | [0.957, 1.01] | 0.131 |
| Baseline Anemia | 1.02 | [0.925, 1.13] | 0.674 |
| Hypoglycemia Frequency | 1.08 | [1.05, 1.11] | **<0.0001** |
| HbA1c (n=20874) |  |  |  |
| Baseline | 1.00 | [0.978, 1.03] | 0.874 |
| Mean | 0.937 | [0.902, 0.973] | **0.001** |
| Standard Deviation | 1.09 | [1.04, 1.15] | **0.001** |
| Coefficient of Variation | 1.01 | [1.005, 1.02] | **<0.0001** |
| Total Cholesterol (n=18926) |  |  |  |
| Baseline | 1.02 | [0.982, 1.07] | 0.264 |
| Mean | 0.872 | [0.820, 0.927] | **<0.0001** |
| Standard Deviation | 1.18 | [1.08, 1.28] | **<0.0001** |
| Coefficient of Variation | 1.016 | [1.01, 1.02] | **<0.0001** |
| HDL Cholesterol (n=17930) |  |  |  |
| Baseline | 0.965 | [0.852, 1.09] | 0.573 |
| Mean | 0.666 | [0.566, 0.783] | **<0.0001** |
| Standard Deviation | 1.82 | [1.09, 3.05] | **0.022** |
| Coefficient of Variation | 1.024 | [1.02, 1.03] | **<0.0001** |
| LDL Cholesterol (n=17485) |  |  |  |
| Baseline | 0.975 | [0.924, 1.03] | 0.343 |
| Mean | 0.792 | [0.734, 0.854] | **<0.0001** |
| Standard Deviation | 1.31 | [1.14, 1.51] | **<0.001** |
| Coefficient of Variation | 1.015 | [1.01, 1.02] | **<0.0001** |
| Triglyceride (n=18889) |  |  |  |
| Baseline | 0.975 | [0.944, 1.01] | 0.123 |
| Mean | 1.04 | [1.01, 1.07] | **0.018** |
| Standard Deviation | 0.996 | [0.954, 1.04] | 0.850 |
| Coefficient of Variation | 0.998 | [0.995, 1.00] | 0.218 |
| Anti-Diabetic Agent |  |  |  |
| Sulphonylurea | 1.10 | [1.00, 1.21] | **0.045** |
| Biguanide | 0.682 | [0.621, 0.748] | **<0.0001** |
| DPP4 Inhibitor | 0.585 | [0.262, 1.30] | 0.190 |
| Thiazolidinedione | 0.816 | [0.632, 1.05] | 0.119 |
| Alpha-Glucosidase Inhibitor | 0.972 | [0.743, 1.27] | 0.836 |
| Cardiovascular Medications |  |  |  |
| ACEI/ARB | 1.62 | [1.46, 1.80] | **<0.0001** |
| Beta-blocker | 2.24 | [2.04, 2.46] | **<0.0001** |
| Calcium channel blocker | 2.23 | [2.03, 2.45] | **<0.0001** |
| Diuretic | 2.44 | [2.22, 2.68] | **<0.0001** |
| Lipid-lowering agents | 1.49 | [1.35, 1.63] | **<0.0001** |

**Supplementary Table 4.** Univariable Predictors for Ventricular Tachycardia.

| Predictor | Hazard Ratio (HR) | 95% Confidence Interval (CI) | P-Value |
| --- | --- | --- | --- |
| Age | 1.03 | [1.02, 1.05] | **<0.0001** |
| Categorized Age | 1.43 | [1.26, 1.63] | **<0.0001** |
| Male | 1.93 | [1.44, 2.59] | **<0.0001** |
| Frequency of Baseline Acute Admissions | 1.01 | [1.00, 1.01] | **<0.0001** |
| Number of Concomitant DM Complications | 1.26 | [1.08, 1.46] | **0.003** |
| Number of Distinct Non-DM Comorbidities | 1.06 | [1.04, 1.07] | **<0.0001** |
| Baseline Hemoglobin Count | 0.980 | [0.912, 1.05] | 0.576 |
| Baseline Anemia | 0.992 | [0.743, 1.32] | 0.956 |
| Hypoglycemia Frequency | 0.986 | [0.878, 1.11] | 0.806 |
| HbA1c (n=20874) |  |  |  |
| Baseline | 1.02 | [0.908, 1.06] | 0.599 |
| Mean | 0.941 | [0.842, 1.05] | 0.285 |
| Standard Deviation | 1.13 | [0.973, 1.31] | 0.111 |
| Coefficient of Variation | 1.02 | [1.00, 1.03] | **0.031** |
| Total Cholesterol (n=18926) |  |  |  |
| Baseline | 1.00 | [0.877, 1.13] | 0.954 |
| Mean | 1.02 | [0.822, 1.16] | 0.789 |
| Standard Deviation | 1.29 | [1.05, 1.59] | **0.018** |
| Coefficient of Variation | 1.02 | [1.01, 1.04] | **0.008** |
| HDL Cholesterol (n=17930) |  |  |  |
| Baseline | 1.03 | [0.708, 1.48] | 0.897 |
| Mean | 3.46 | [0.168, 0.497] | **<0.0001** |
| Standard Deviation | 1.52 | [0.335, 6.87] | 0.589 |
| Coefficient of Variation | 1.03 | [1.01, 1.04] | **0.002** |
| LDL Cholesterol (n=17485) |  |  |  |
| Baseline | 0.954 | [0.812, 1.12] | 0.566 |
| Mean | 1.01 | [0.819, 1.25] | 0.923 |
| Standard Deviation | 1.62 | [1.12, 2.35] | **0.010** |
| Coefficient of Variation | 1.01 | [1.00, 1.02] | **0.024** |
| Triglyceride (n=18889) |  |  |  |
| Baseline | 1.02 | [0.963, 1.09] | 0.441 |
| Mean | 1.09 | [1.03, 1.16] | **0.003** |
| Standard Deviation | 1.06 | [0.969, 1.15] | 0.211 |
| Coefficient of Variation | 1.01 | [0.999, 1.01] | 0.088 |
| Anti-Diabetic Agent |  |  |  |
| Sulphonylurea | 1.26 | [0.961, 1.66] | 0.094 |
| Biguanide | 0.494 | [0.377, 0.650] | **<0.0001** |
| DPP4 Inhibitor | / | / | / |
| Thiazolidinedione | 0.831 | [0.391, 1.77] | 0.629 |
| Alpha-Glucosidase Inhibitor | 0.757 | [0.312, 1.84] | 0.539 |
| Cardiovascular Medications |  |  |  |
| ACEI/ARB | 1.41 | [1.05, 1.90] | **0.023** |
| Beta-blocker | 2.56 | [1.93, 3.39] | **<0.0001** |
| Calcium channel blocker | 1.36 | [1.03, 1.78] | **0.029** |
| Diuretic | 3.87 | [2.93, 5.10] | **<0.0001** |
| Lipid-lowering agents | 1.94 | [1.47, 2.57] | **<0.0001** |
